# Two peptides derivate from *Acinetobacter baumannii* outer membrane protein K as vaccine candidates: a comprehensive in silico study

**DOI:** 10.1186/s13104-023-06409-9

**Published:** 2023-06-30

**Authors:** Hana Heidarinia, Elahe Tajbakhsh, Mosayeb Rostamian, Hassan Momtaz

**Affiliations:** 1 Department of Microbiology, Faculty of Basic Sciences, Shahrekord Branch, Islamic Azad University, Shahrekord, Iran; 2 Infectious Diseases Research Center, Health Institute, Kermanshah University of Medical Sciences, Imam Reza Hospital, Parastar Blvd, Kermanshah, 6714415333 Iran

**Keywords:** *Acinetobacter baumannii*, Epitope, In silico, Molecular docking, Outer membrane protein K

## Abstract

**Background:**

The lack of appropriate vaccines is an obstacle to the effective management of *A. baumannii* infections. Peptide vaccines offer an attractive and promising preventive strategy against *A. baumannii*.

**Objective:**

In this study, we identified specific T cell epitopes of *A. baumannii* outer membrane protein K (OMPK) using comprehensive bioinformatics and detailed molecular docking analysis.

**Methods:**

Both class-I and class-II T cell epitopes of *A. baumannii* OMPK were predicted by three tools namely IEDB, SYFPEITHI, and ProPred. The predicted epitopes were shortlisted based on several analyses including prediction scoring, clustering, exclusion of human similarity, considering immunogenicity and cytokine production, and removal of toxic and/or allergen epitopes. The epitopic peptides with high prediction scores and appropriate properties containing both class-I and class-II T cell epitopes were selected. Two of these class I/II epitopic peptides were chosen for molecular docking studies and assessing their physicochemical properties as vaccine candidates.

**Results:**

The results showed many T-cell epitopes of OMPK that could be evaluated for possible immunogenicity. Two of these epitopes (containing both class-I and II epitopes) had high prediction scores, were predicted by several tools, attached to several HLAs, and had the best docking score. They had different physicochemical properties and were conserved among Acinetobacter species.

**Discussion:**

We identified the *A. baumannii* OMPK high immunogenic class-I and class-II T cell epitopes and introduced two promising high immunogenic peptides as vaccine candidates. It is recommended to perform in vitro/in vivo investigation of these peptides to determine their true efficacy and efficiency.

**Supplementary Information:**

The online version contains supplementary material available at 10.1186/s13104-023-06409-9.

## Background

Bacteria of the genus Acinetobacter are non-motile Gram-negative coccobacilli that are ubiquitous in nature but are mainly found in soil, water, and sewage [1]. *Acinetobacter baumannii*, an opportunistic pathogen responsible for hospital-acquired infections, is considered a serious threat to public health [2]. This bacterium causes pneumonia, urinary tract infection, blood infection, secondary meningitis, and wound infection [3]. In intensive care units (ICUs), *A. baumannii* is responsible for approximately 20.9% of all hospital-acquired infections in Europe, the Eastern Mediterranean, and Africa [4]. Hospital-acquired pneumonia is one of the most common clinical manifestations of *A. baumannii* infections and occurs mostly in patients receiving mechanical ventilation in ICUs [5]. A recent meta-analysis of 126 studies from 29 countries worldwide showed that multidrug-resistant *A. baumannii* was present in 79.9% of all cases of acquired pneumonia and ventilator-associated pneumonia (VAP) [6].

*Acinetobacter baumannii* can be spread through respiratory droplets such as respiratory secretions, sneezes, and saliva, as well as through person-to-person contact, skin picking, and contact with contaminated surfaces [7]. *A. baumannii* ability to propagate easily is mainly due to three characteristics: desiccation resistance, the ability to form biofilms on nonliving surfaces, and a propensity to adhere to host cells [8]. The increase in drug-resistant strains of *A. baumannii* has made it difficult to control using common antibiotics. Therefore, the development of effective vaccines is an alternative means to prevent infections caused by *A. baumannii* [7]. As a result of antibiotic shortages, the World Health Organization has designated *A. baumannii* as a key priority for the development of new medical countermeasures, including vaccine development [9].

Over the past decade, research efforts have identified several promising immunomodulatory strategies that could lead to a safe and effective vaccine against *A. baumannii* [9]. Several vaccine candidates have been tested for their ability to immunize model animals against *A. baumannii* infection. However, none of them have yet received approval for use in humans, and further research is still needed to develop a suitable vaccine candidate against *A. baumannii* [10].

Outer membrane proteins^1^ (OMPs) play a decisive role in antibiotic resistance and the pathogenicity of *A. baumannii* [11]. One of the challenges in targeting the outer membrane components of *A. baumannii* is the presence of a dense polysaccharide capsule that shields most of the outer membrane antigens from immune recognition [12]. OMPs are abundant in the outer membrane of bacteria and often protrude from the polysaccharide capsule, making them a suitable target for vaccines due to their ability to induce a strong antibody response, primarily IgG [13, 14]. The most effective *A. baumannii* vaccines are often composed of antigens present on the bacterial outer membrane, such as OMPA, OMP22, and OMPK [9].

Outer membrane protein K^2^ (OMPK), also known as TSX, is a 241 amino acid *A. baumannii* OMP that is specific for the nucleoside-forming ion channels. This protein is equivalent to OMPK in the fish pathogen *Vibrio harveyi* [15]. Previous studies on animal models have demonstrated that *A. baumannii* OMPK has significant immunogenic properties and is considered a promising vaccine candidate [15–17]. Both B cell and T cell responses seem to be involved in protection against *A. baumannii*, although no accurate information about OMPK epitopes vaccine potential can be found in the literature.

The development of epitopic (peptide) vaccines could offer an attractive and viable treatment option for *A. baumannii*-related diseases. Today, in addition to molecular approaches, computer calculations and bioinformatics studies have provided a new method for vaccine creation through in silico epitope prediction. Numerous studies, similar to the present study, have utilized bioinformatics and sometimes laboratory analysis to investigate suitable vaccine candidates for various bacteria [18–23]. The present study aims to identify specific T-cell epitopes of *A. baumannii* OmpK through comprehensive bioinformatics and detailed molecular docking analysis, based on cell-dependent immune responses. In addition, this study introduces two T-cell epitopic peptides derived from OMPK, which exhibit suitable immunological and physicochemical properties, as potential *A. baumannii* vaccine candidates.

## Methods

### OMPK sequence

The sequence of *A. baumannii* OMPK was retrieved from the protein database of NCBI (https://www.ncbi.nlm.nih.gov/protein, accession number: CRL96222.1).

### T cell epitopes prediction

The most frequent human leukocyte antigen^3^ (HLA)-I (specific for class-I or CD8+ T cell-specific epitopes) and HLA-II (specific for class-II or CD4+ T cell-specific epitopes) alleles were determined using the Allele frequencies server (http://www.allelefrequencies.net/), the prediction servers, or using our previous experiences on *K. pneumoniae* antigens [24–26] (Additional file 1: Table S1).

Similar to our previous studies [24, 27], the prediction of T cell epitopes was carried out using several epitope prediction tools including SYFPEITHI (http://www.syfpeithi.de/bin/MHCServer.dll/EpitopePrediction.htm), IEDB (http://tools.iedb.org), and ProPred (http://crdd.osdd.net/raghava/propred/). For the prediction of class-I and class-II epitopes, the percentile ranks of ≤ 1 and ≤ 10, respectively, were selected as the thresholds in IEDB. In ProPred-I and SYFPEITHI servers, the score of ≥ 10 was used as the threshold, whereas in ProPred-II all the predicted epitopes were chosen. Epitopes of 9 and 15 residues were chosen for the prediction of class-I and class-II epitopes, respectively.

Regarding ≥ 70 similarities, the predicted epitopes were clustered with the IEDB clustering tool (http://tools.iedb.org/cluster/), and an epitope of each cluster was selected for advanced analyses based on its higher prediction score compared to the others. The immunogenicity of class-I epitopes was predicted by a specific IEDB tool (http://tools.iedb.org/immunogenicity/). The potency of class-II epitopes in interferon-gamma^4^ (IFN-γ) production was estimated by the IFNepitope server (http://crdd.osdd.net/raghava/ifnepitope/).

### Class II/class I windows and final T cell epitopes

The class-II/class-I epitopic windows were found by the IEDB clustering tool by putting the cut-off at ≥ 70% similarity. The class-II epitopes possessing at least one class-I epitope were chosen.

The final selection of T-cell epitopes was based on several criteria, including the epitopes predicted by multiple tools, those comprising both class-I and class-II epitopes, those with higher prediction scores, and class-II epitopes with IFN-γ production ability or class-I epitopes with higher immunogenicity scores.

### Toxicity, human similarity, allergenicity, and experimental records

The T cell epitopes were assessed for possible toxicity using the ToxinPred server (https://webs.iiitd.edu.in/raghava/toxinpred/algo.php). The percentage of human similarity was estimated by two indicators of coverage and identity in the BLASTP server using the human proteome (taxid 9606) as the reference organism. The ≥ 90% similar epitopes to human proteome were excluded from further analyses. The AllerCatPro [28] and the Structural Database of Allergenic Proteins^5^ (SDAP) [29] tools, were used to predict possible allergenicity.

Epitopes were searched for any experimental record using the IEDB home page (https://www.iedb.org/).

### Tertiary structures

The structure of the OMPK protein was generated using the SWISS-MODEL server (https://swissmodel.expasy.org/, [30]), with the experimentally determined structure of the *E. coli* OMPK protein (ID: 1TLW) used as a template.

The PEP-FOLD server [31] was applied to predict the tertiary structure of the epitopes. The structure of selected final HLA alleles was retrieved from the PDB databank with the following IDs: 5HHP for HLA-A*02:01 and 1BX2 for HLA-DRB1*15:01.

The accuracy of the protein structure model was evaluated using ERRAT (https://saves.mbi.ucla.edu/, [32]), the Z-score plot provided by the ProSA-web server (https://prosa.services.came.sbg.ac.at/prosa.php, [33]), and the Ramachandran plot generated by the Molprobity server (http://molprobity.biochem.duke.edu/, [34]).

### Molecular docking studies

For binding of class-I and -II epitopes to their HLA alleles, molecular docking studies were performed by HADDOCK 2.2 tool (http://haddock.science.uu.nl/services/HADDOCK2.2). Consensus prediction of interface residues in transient complexes (CPORT) (https://milou.science.uu.nl/services/CPORT/) was applied to accurate prediction of amino acid residues incorporated in the binding [35]. Docking was performed and the results were reported in terms of HADDOCK scores along with some secondary scores. As controls, the original ligands of HLA PDB files that were previously characterized were utilized.

### Prediction of physicochemical characteristics, structure analyses, and antigenicity

The ProtParam tool (https://web.expasy.org/protparam/) [36] was used to estimate the physicochemical characteristics of the epitopes including molecular weight, isoelectric pH^6^ (pI), grand average of hydropathicity index^7^ (GRAVY), instability index, and the estimated half-life. The net charge and water solubility of the epitopes were predicted by PepCalc (https://pepcalc.com/) and the epitopes’ hydrophobicity was predicted by PEPTIDE 2.0 (https://www.peptide2.com/N_peptide_hydrophobicity_hydrophilicity.php). Epitope conservancy was checked via BLASTP and NCBI conserved domain search.

The secondary structure prediction was done by the Stride Web interface (http://webclu.bio.wzw.tum.de/cgi-bin/stride/stridecgi.py). The structures and location of the epitopes were depicted by the Web3DMol server (http://web3dmol.net/). The simplified molecular-input line-entry system^8^ (SMILES) string of the peptides was obtained from the PepSMI server (https://www.novoprolabs.com/tools/convert-peptide-to-smiles-string) and used to depict 2D structures of the peptides.

Antigenicity prediction of the final epitopes was carried out by the Vaxijen v2.0 (http://www.ddg-pharmfac.net/vaxijen/VaxiJen/VaxiJen.html, [37]), and ANTIGENpro (https://scratch.proteomics.ics.uci.edu/) web servers.

## Results

### Prediction of T cell epitopes

T cell epitopes were predicted and several analyses were conducted to screen the predicted epitopes as follows: (1) excluding duplicate epitopes and choosing one epitope from an epitope cluster, (2) choosing high-scored epitopes and/or the epitopes predicted by more prediction tools, (3) weighing positivity in IFN-γ production and higher immunogenicity score for class-II and class-I epitopes, respectively, as preferred criteria of epitope selection, and (4) excluding allergens, toxic epitopes, and the epitopes with human similarity of 90% or more. By employing these analyses, the dominant class-I and class-II epitopes were chosen, and also their class II/I windows were found by comparing both classes with each other.

The number of initially predicted class-I and class-II epitopes were 608, and 441, respectively, which were reduced to 92 and 62 following clustering and 22 and 27 after screening strategies (Table 1).

**Table 1:** The number of predicted T cell epitopes of *A. baumannii* OMPK before and after clustering and screening

Prediction tool	Class-I				Class-II			
	IEDB	SYFPEITHI	ProPred-I	Total	IEDB	SYFPEITHI	ProPred-II	Total
Before clustering	85	477	46	608	118	307	16	441
After clustering	28	37	27	92	13	41	8	62
After screening^a^	–	–	–	22	–	–	–	27

^a^Screening include excluding duplicates, toxic, allergens, and human peptides similar epitopes and choosing high-scored, more frequently predicted, potent in IFN-γ production, and immunogenic epitopes

### The characteristics of short-listed epitopes

None of our predicted epitopes was toxic or allergen. Also, none of our selected epitopes had ≥ 90% similarity with the human peptides. In addition, no previous experimental records were found on the epitopes.

### The characteristics of class II/I epitopic windows

Five class II/I epitopic windows were found (Table 2). The table also presents the prediction scores of each epitope by each prediction tool, as well as information on toxicity, allergenicity, and human similarity of the epitopes. Two class-II/class-I windows, named Ep1 and Ep2, were selected for further studies.

**Table 2:** The class II/I epitopic windows of *A. baumannii* OMPK

Class II/I epitopic window^b^	Class-II epitopes										
	Epitope	HLA allele	Score^a^								
			SYFPEITHI	ProPred-II	IEDB	IFNg	Toxicity	Allergenicity	Docking		Human similarity (coverage %, identity %)
									Test	Control	
DYQMT**FVYGIPFKI**A	DYQMTFVYGIPFKIA	DRB1*15:01	20	N/A	7.3	+	–	–	− 23.6 ± 16.1	− 21.4 ± 13.1	100, 62.5
QL**KQLAATCAL**LSAT	QLKQLAATCALLSAT	DRB1*07:01	14	N/A	8.5	+	–	–	N/A	N/A	100, 45
AKV**KYADVFFFM**DRM	AKVKYADVFFFMDRM	DRB1*15:01	10	N/A	N/A	+	–	–	N/A	N/A	86, 57.1
**IPYFQYANL**NFYRAN	IPYFQYANLNFYRAN	DRB1*15:01	N/A	+	4.6	–	–	–	N/A	N/A	40, 64.2
GKH**ISPDTRLYL**GIE	GKHISPDTRLYLGIE	DRB1*15:01	20	N/A	N/A	–	–	–	− 39.7 ± 16.1	− 41.4 ± 15.1	86, 53.8

Class II/I epitopic window^b^	Class-I epitopes										
	Epitope	HLA allele	Score^a^								
			SYFPEITHI	ProPredI	IEDB	Immunogenicity	Toxicity	Allergenicity	Docking		Human similarity (coverage %, identity %)
									Test	Control	
DYQMT**FVYGIPFKI**A	FVYGIPFKI	A*02:01	18	12	0.03	0.13	–	–	− 20.9 ± 14.0	− 22.4 ± 11.0	88, 75
QL**KQLAATCAL**LSAT	KQLAATCAL	A*02:01	16	28	N/A	0.09	–	–	N/A	N/A	100, 77.8
AKV**KYADVFFFM**DRM	KYADVFFFM	A*24:02	100	N/A	0.01	0.35	–	–	N/A	N/A	88, 58.3
**IPYFQYANL**NFYRAN	IPYFQYANL	B*35:01	20	20	0.64	0.03	–	–	N/A	N/A	77, 77.8
GKH**ISPDTRLYL**GIE	ISPDTRLYL	A*02:01	14	N/A	N/A	0.09	–	–	− 22.5 ± 7.8	− 25.0 ± 7.8	100, 77.8

Only the highest scores obtained from prediction tools are presented here. Also, only the most repeated HLA allele is presented for epitopes

*N/A* not available

^a^For IEDB, the lower percentile rank (number) means the higher score (thresholds: 1 for class-I and 10 for class-II epitopes); for SYFPEITHI and ProPred-I, the higher scores are shown by the higher numbers (thresholds are equal to 10 for both tools); for ProPred-II, the “+” indicates that the epitope is predicted by the tool; in immunogenicity, the more immunogenic epitope is shown by the more positive number; in IFNg column, the “+” shows that the epitope is able to produce IFNg; in Toxicity and Allergenicity columns, being not toxic or allergen is presented by the “–”; in Docking, HADDOCK scores (kcal/mol) are presented as affinity ± standard deviation (test is our complex and control is the complex of HLA with its original peptides); in Human similarity, the numbers show the highest percentage of coverage and identity values reported by BLASTP server

^b^In this column, the regions containing both class-II and class-I epitopes (the epitopic windows) are presented. The bold fonts show the class I epitope residing in the class II epitope

### Evaluation of protein/epitopes tertiary structures

**Fig. 1:**
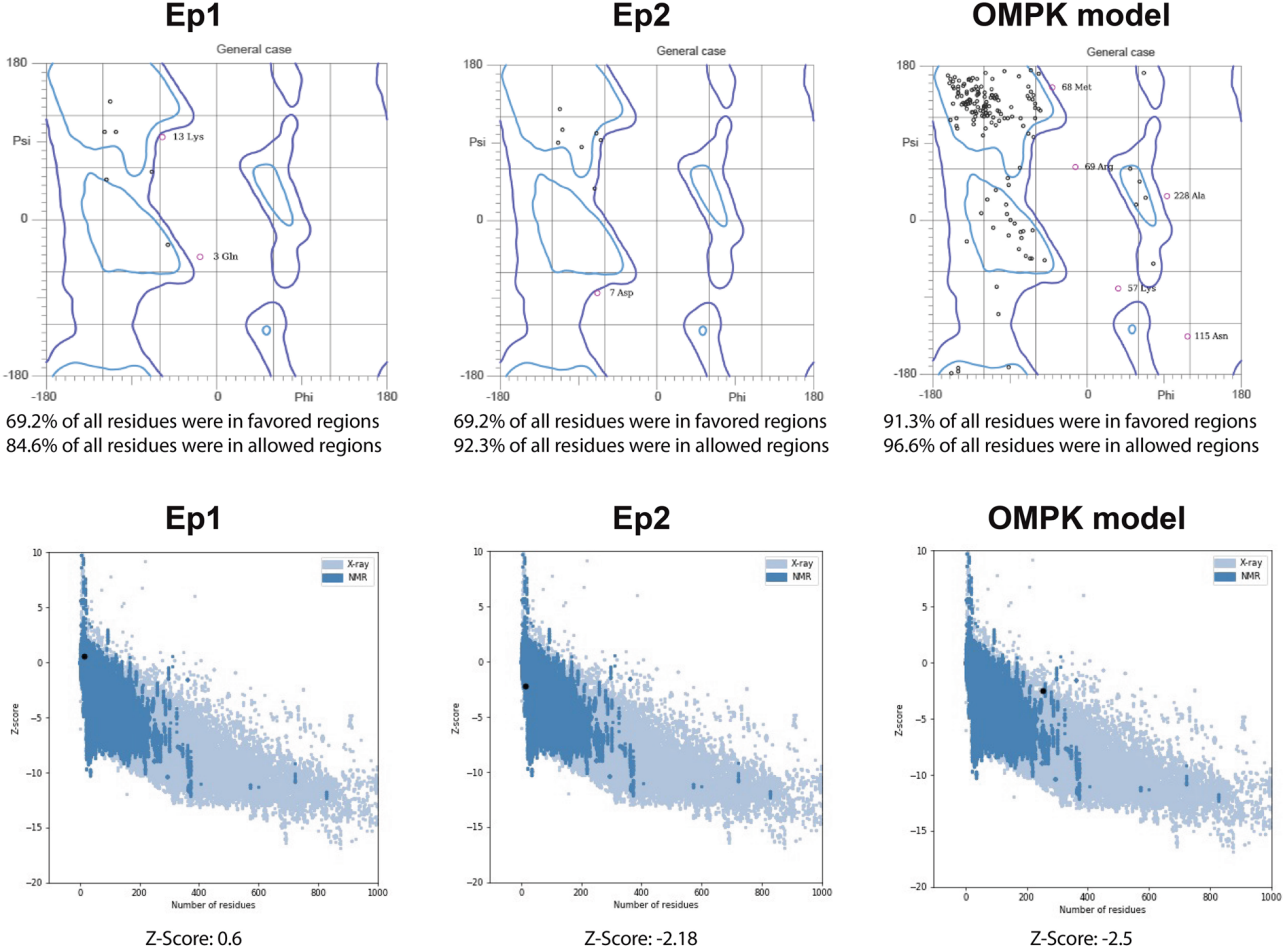
Structural quality validation of *A. baumannii* OMPK and its final peptides derivate (Ep1 and Ep2). The upper parts of the figure show the Ramachandran plots of the modeled structures of Ep1, Ep2, and the full OmpK protein. The number of residues located in the favored and allowed regions of the Ramachandran plots are shown below each plot. The lower parts of the figure present the ProSA-web Z-score plots. These plots demonstrate that the models of Ep1, Ep2, and the full OmpK protein (represented by black points) all fall within the range of scores typically observed for native proteins of similar size (represented by colored regions). The Z-score of each model is also shown below its corresponding plot

The tertiary structures of the OMPK protein and the final epitopes (Ep1 and Ep2) were modeled. The overall quality factor for Ep1, Ep2, and OMPK, as determined by ERRAT, was 79.21, 73.13, and 89.67, respectively. The Ramachandran plot showed that most amino acid residues in all three molecules (Ep1, Ep2, and OMPK) were located in the allowed and/or favored regions (Fig. 1). Moreover, the Z-scores for these molecules were 0.6, − 2.18, and -2.5 respectively, which fell within the range of scores typically observed for native proteins of similar size from different sources (X-ray, NMR) (Fig. 1).

### Molecular docking results

Class-II epitopes located in Ep1 and Ep2 were predicted to bind HLA-DRB1*15:01. Similarly, class-I epitopes were bound to HLA-A*02:01.

After retrieving the 3D structures of epitopes (ligands) and HLA molecules (receptors), ligand-receptor docking was performed by the HADDOCK tool. The docking scores of our class I epitope-HLA and class II epitope-HLA were higher than the docking scores of HLA combined with their original peptides (controls), showing the high affinity of our epitopes to their specific HLA molecules (Table 2).

### The structure, antigenicity, and physicochemical properties of the final peptides

Table 3 presents the physicochemical properties of the two final epitopes (peptides). Both peptides consist of 15 amino acid residues. The Ep1 (DYQMTFVYGIPFKIA) peptide (1793.11 Dalton) had 53.33% hydrophobicity and a pI of 5.83. Its estimated half-life was 1.1 h in mammalian reticulocytes in vitro and > 10 min in *E. coli*, in vivo. The peptide is poorly water-soluble, but it is stable with a net charge of zero. The Ep2 (GKHISPDTRLYLGIE) peptide (1698.94Dalton) had 33.33% hydrophobicity and a pI of 6.75. Its estimated half-life was > 30 h in mammalian reticulocytes in vitro and > 10 min in *E. coli*, in vivo. The peptide is good water-soluble, but it is unstable with a net charge of 0.1.

**Table 3:** The properties of final peptides derivated from* A. baumannii* OMPK

Peptide name	Sequence	Number of amino acid residues	Molecular weight (Da)	Hydrophobicity (%)	Estimated water solubility	Net charge at pH = 7.0	pI	GRAVY	Instability index	Estimated half-life	Antigenicity^a^	
											Vaxijen score	ANTIGENpro score
Ep1	DYQMTFVYGIPFKIA	15	1793.11	53.33	Poor	0	5.83	0.42	− 5.47, stable	1.1 h (mammalian reticulocytes, in vitro)	1.75 (antigen)	0.046 (non-antigen)
										3 min (yeast, in vivo)		
										> 10 min (*Escherichia coli*, in vivo)		
Ep2	GKHISPDTRLYLGIE	15	1698.94	33.33	Good	0.1	6.75	− 0.5	70.85, unstable	30 h (mammalian reticulocytes, in vitro)	0.42 (antigen)	0.077 (non-antigen)
										> 20 min (yeast, in vivo)		
										> 10 min (*Escherichia coli*, in vivo)		

*GRAVY* grand average of hydropathicity index, *pI* isoelectric pH

^a^Antigenicity was assessed by Vaxijen and ANTIGENpro. The threshold of Vaxijen was 0.4, so Ep1 and Ep2 which had higher scores were predicted as antigens. In ANTIGENpro, whatever the scores close to 1.0 they were assumed as more probable antigens. None of our epitopes had a high ANTIGENpro score, hence predicted as non-antigen

Both Ep1 and Ep2 peptides were predicted to be antigens by the Vaxijen server, while neither of them was predicted to be an antigen by the ANTIGENpro server (Table 3).

**Fig. 2:**
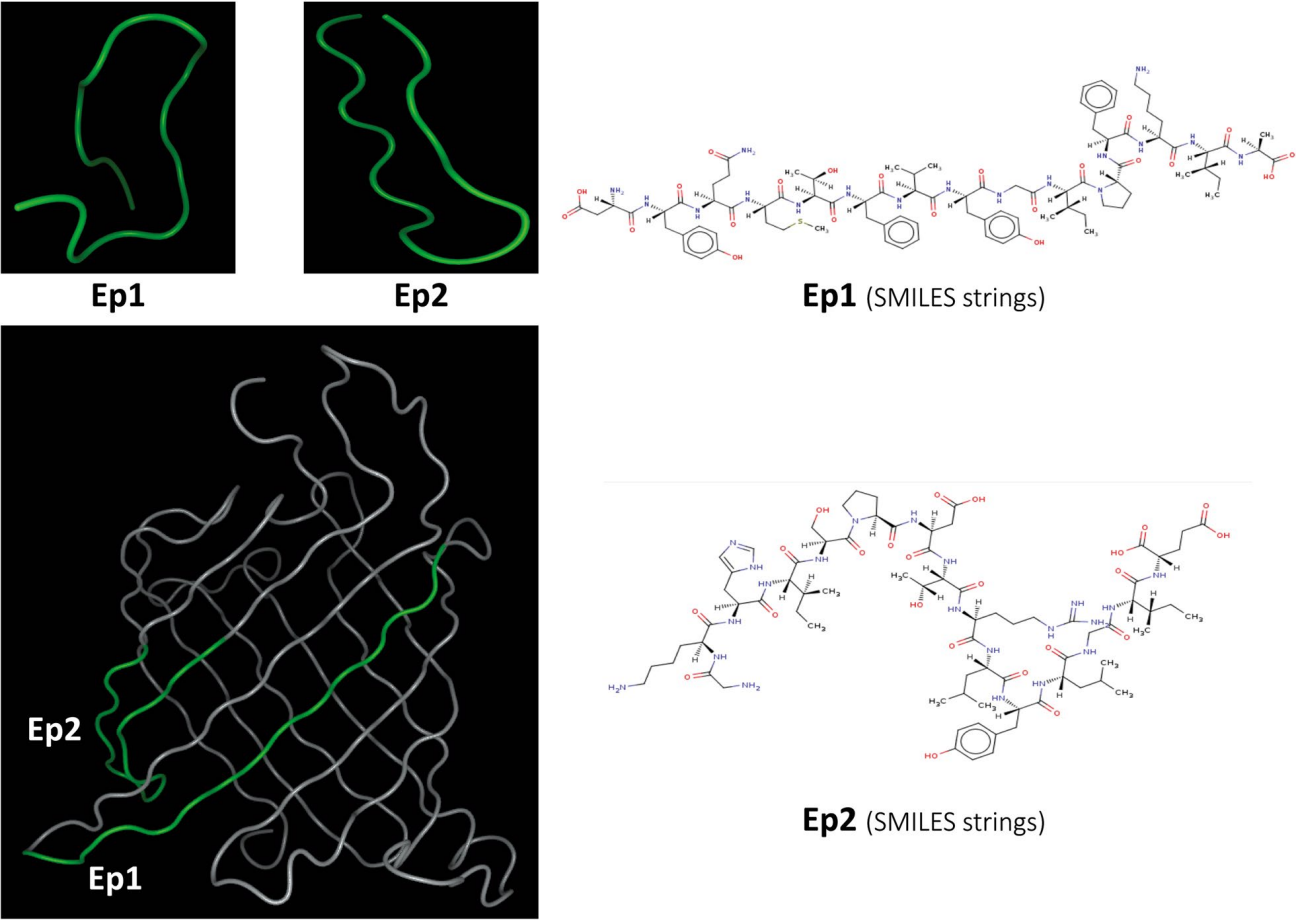
The structure and location of final peptides derivate (Ep1 and Ep2) from *A. baumannii* OMPK. The 3D structure of Ep1 (DYQMTFVYGIPFKIA) and Ep2 (GKHISPDTRLYLGIE) peptides, along with their corresponding simplified molecular-input line-entry system (SMILES) strings and locations in the 3D structure of OMPK, are presented. As shown, both Ep1 and Ep2 are located on the lateral surfaces of the OMPK structure

Both peptides are mainly composed of turns and they were both located on the lateral surfaces of the OMPK structure (Fig. 2). The peptides were conserved among Acinetobacter species, but not among other species except ion channel protein TSX of *Klebsiella pneumoniae*.

## Discussion

Previous research on OMPs has shown that these proteins can trigger defense against *A. baumannii* infections through both cell-mediated and humoral immunity. OMPK was able to trigger both humoral (IgG antibodies) and cytokines responses, which provided defense against a fatal challenge with *A. baumannii* [15, 16, 38]. The efficiency of OMPK vaccination was improved when fused with another OMP, called OMP22, or when adjuvanted with MF59 [15, 16]. MF59 is an oil-in-water emulsion adjuvant that has been shown to elicit innate immunity, vaccine-specific IgG antibodies, and effector CD8 T cells [39–41]. In addition to MF59, various other adjuvants have been developed that can be used to boost immunogenicity. One such example is Toll-like receptor (TLR) agonists, which have been shown to possess potent adjuvant activity in our previous studies and others [42–46].

Host defense against *A. baumannii* infections likely involves significant contributions from humoral immune responses, including IgM, IgG, and IgA [47]. The T cell immune responses to *A. baumannii* infections are still poorly understood. According to a small number of experimental studies, *A. baumannii* infection severity was linked to higher levels of Th1 inflammatory cytokine responses and lower levels of IL-10, suggesting a protective effect of Th2 immune responses [48, 49]. However, the precise functions of Th1 and Th2 in host defense against *A. baumannii* infections are still unknown [50], demanding more research in this field. In the present study, we found two potent T cell epitopic peptides (Ep1 and Ep2) that possess many favorable properties as *A. baumannii* vaccine candidates. They were both immunogenic, non-toxic, non-allergen, and non-similar to the human proteome. In addition, they contain both class I and II T cell epitopes that were found to bind to their HLAs with high affinity. Ep1 was predicted to be able to produce IFN-γ while Ep2 could not, but it should not be assumed as a negative criterion since as mentioned above, the role of Th1 cytokines such as IFN-γ is not clearly understood in protection against *A. baumannii* [50].

In the present study, the modeled 3D structures underwent verification using in silico tools. The majority of amino acid residues in our modeled protein (OMPK) and its final epitopic regions (Ep1 and Ep2) were located in the allowed/favored regions of the Ramachandran plot, indicating the high quality of our models. Furthermore, we utilized the ERRAT assessment to identify any potential anomalies in the structure [32], which showed a high-quality factor for our model. A factor closer to 100 in the ERRAT assessment indicates a better model quality. Similarly, the quality of our models was demonstrated by the Z-score, which is correlated with experimentally determined X-ray and/or NMR structures [33].

The results of the present study showed that Ep1 and Ep2 peptides have different physicochemical properties so Ep1 is stable, poor water-soluble, more hydrophobic, and with a lower estimated half-life than Ep2. Being more hydrophilic is indicated by the negative GRAVY [36], as seen for Ep2 but not Ep1. The pI index is a crucial consideration when choosing a vaccine candidate and shouldn't fall within the range of body tissues’ pH (7.2–7.6) [51]. The pI of both Ep1 and Ep2 peptides was outside of the pH range of body tissues. These parameters suggested both peptides as potential vaccine candidates for more in vitro and in vivo studies. Notably, the properties of these peptides can be improved using various chemical/biochemical techniques. Furthermore, these epitopes can be incorporated into the design of multi-valent and multi-epitope vaccine candidates, which can potentially be more stable and effective. Previous studies have employed similar strategies for vaccination against *A. baumannii* [20, 23, 52, 53].

Both epitopes identified in this study are located on the surface of the OMPK protein, making them easily accessible to antibodies. This feature, in addition to their potential as vaccine candidates, also makes them suitable candidates for the diagnosis of *A. baumannii*-related diseases. The epitopes may be chemically synthesized or cloned/expressed and utilized as radiochemical probes in designing diagnostic kits [54].

The conservancy study of the Ep1 and Ep2 peptides showed that they do not belong to a conserved domain superfamily, but they are highly conserved among Acinetobacter species, hence it may induce immune responses against many species of this genus. Also, both peptides had 100% similarity to a sequence of ion channel protein TSX of *K. pneumoniae*, so they may induce immune responses against *K. pneumoniae* as well.

In recent years, the number of bioinformatics studies on *A. baumannii* vaccines has increased significantly. OMPA [55, 56], DcaP [57], NucAb [58], and TonB-dependent copper receptor [59] can be mentioned among the OMP antigens of *A. baumannii* that have been studied in silico. Collectively, the results of our study, along with previous research provide promising prospects for the development of a suitable vaccine based on OMPs to combat *A. baumannii* infections in the future.

Altogether, we identified the *A. baumannii* OMPK high immunogenic class-I and class-II T cell epitopes, which can be utilized in future research. Additionally, two high immunogenic peptides containing both T cell epitope classes as well as the necessary physicochemical properties for the development of an *A. baumannii* vaccine were introduced. It is recommended to do an in vitro/in vivo investigation of these peptides to determine their true efficacy and efficiency.

## Limitations

Limited immunity due to several factors such as easy degradation and their recognition difficulty is linked to epitope-based vaccinations, such as our suggested Ep1 and Ep2 peptides. Conjugating them with conventional adjuvants and/or novel built-in adjuvants, such as new biomaterials or carriers, is one efficient technique to bypass this limitation [60]. Another limitation of our suggested vaccine candidate peptides is that they will primarily be given to older and immunocompromised individuals that are not able to mount strong immune responses [61].

## Supplementary Information

**Additional file 1: Table S1.** The list of HLA alleles used for *A. baumannii* OMPK T cell epitope prediction.

## Data Availability

All data generated or analyzed during this study are included in this published article.

## Footnotes

1 OMPs: outer membrane proteins.

2 OMPK: outer membrane protein K.

3 HLA: human leukocyte antigen.

4 IFN-γ: interferon-gamma.

5 SDAP: Structural Database of Allergenic Proteins.

6 pI: isoelectric pH.

7 GRAVY: grand average of hydropathicity index.

8 SMILES: simplified molecular-input line-entry system.
